# Early Findings in Neonatal Cases of *RYR1*–Related Congenital Myopathies

**DOI:** 10.3389/fneur.2021.664618

**Published:** 2021-06-28

**Authors:** Eleonora Mauri, Daniela Piga, Alessandra Govoni, Roberta Brusa, Serena Pagliarani, Michela Ripolone, Robertino Dilena, Claudia Cinnante, Monica Sciacco, Denise Cassandrini, Vincenzo Nigro, Nereo Bresolin, Stefania Corti, Giacomo P. Comi, Francesca Magri

**Affiliations:** ^1^Neurology Unit, Foundation IRCCS Ca' Granda Ospedale Maggiore Policlinico, Milan, Italy; ^2^Neuroscience Section, Dino Ferrari Centre, Department of Pathophysiology and Transplantation (DEPT), University of Milan, Milan, Italy; ^3^Neuromuscular and Rare Diseases Unit, Istituto di Ricerca e Cura a Carattere Scientifico Foundation Ca' Granda Ospedale Maggiore Policlinico, Milan, Italy; ^4^Neuropathophysiology Unit, Istituto di Ricerca e Cura a Carattere Scientifico Foundation Ca' Granda Ospedale Maggiore Policlinico, Milan, Italy; ^5^Neuroradiology Unit, Istituto di Ricerca e Cura a Carattere Scientifico Foundation Ca' Granda Ospedale Maggiore Policlinico, Milan, Italy; ^6^Molecular Medicine, Istituto di Ricerca e Cura a Carattere Scientifico Fondazione Stella Maris, Pisa, Italy; ^7^“Luigi Vanvitelli” University and Telethon Institute of Genetics and Medicine (TIGEM), Naples, Italy

**Keywords:** congenital myopathy, *RyR1*, fetal brain MRI, neonatal, muscle MRI, muscle biopsy

## Abstract

Ryanodine receptor type 1-related congenital myopathies are the most represented subgroup among congenital myopathies (CMs), typically presenting a central core or multiminicore muscle histopathology and high clinical heterogeneity. We evaluated a cohort of patients affected with Ryanodine receptor type 1-related congenital myopathy (*RYR1*-RCM), focusing on four patients who showed a severe congenital phenotype and underwent a comprehensive characterization at few months of life. To date there are few reports on precocious instrumental assessment. In two out of the four patients, a muscle biopsy was performed in the first days of life (day 5 and 37, respectively) and electron microscopy was carried out in two patients detecting typical features of congenital myopathy. Two patients underwent brain MRI in the first months of life (15 days and 2 months, respectively), one also a fetal brain MRI. In three children electromyography was performed in the first week of life and neurogenic signs were excluded. Muscle MRI obtained within the first years of life showed a typical pattern of *RYR1*-CM. The diagnosis was confirmed through genetic analysis in three out of four cases using Next Generation Sequencing (NGS) panels. The development of a correct and rapid diagnosis is a priority and may lead to prompt medical management and helps optimize inclusion in future clinical trials.

## Introduction

Ryanodine receptor type 1-related congenital myopathies (*RYR1*-RCM) are the most represented subgroup among congenital myopathies (CM) and are associated with mutations in *RYR1* (1–3). *RYR1* is a large gene encoding calcium-(Ca2+) channel (RyR1), which is a homotetrameric protein embedded in the sarcoplasmic reticulum membrane of skeletal muscle (4, 5). Mutations in *RYR*1 can be extremely heterogeneous even in patients showing the same genotype (6–8). *RYR1*-RCM manifests with symptoms ranging from perinatal onset with floppy baby syndrome to late-onset muscular weakness with slow evolution (2, 9). Both dominant and recessive inheritance have been reported, the latter associated with a more severe clinical phenotype with arthrogryposis and muscular weakness in proximal lower limb muscles (10, 11). In cases with childhood onset, motor milestones are typically delayed. Ptosis, hip dislocation, scoliosis, and tendon retractions are common. The disease course is often favorable (12).

The ongoing classification of CM is based on muscle biopsy features (1, 2). Typical findings at muscle biopsy are central core, minicore, centronuclear myopathy, and congenital fiber type disproportion (CFTD) with type I predominance (3). Muscle histology sometimes may be unspecific or can even be misleading since cores may not be appreciable in the very early stages of the disease and some muscles (i.e., rectus femoris) may not show pathological alterations (3, 13, 14). Moreover, Helbling et al. described cases of *RYR1* mutations associated with dystrophic changes at biopsy and normal immunostaining for proteins associated with congenital muscular dystrophies (15).

In 2004 Jungbluth et al. proposed the use of muscle Magnetic Resonance Imaging (MRI) to describe specific patterns of muscle involvement related to different genes mutated in CM (16), suggesting that MRI patterns correlate with the gene mutation more specifically than histopathology. Mutations in *RYR1* are associated with the most repetitive muscle MRI pattern among CM, the gluteus maximus being the most involved muscle of the pelvis along with adductor magnus, vasti lateralis and intermedius, semitendinosus, and sartorius in the tight. Rectus femoris, biceps femoris, gracilis and adductor longus are usually spared (17).

Even though early onset CM has been largely reported in literature, few cases underwent an early comprehensive characterization through histopathology, electromyography (EMG), and muscle MRI at the very first stages of life (3, 18–20). Therefore, we analyzed our cohort of *RYR1*-mutated patients, focusing on neonatal cases who had undergone muscle biopsy, EMG, and muscle MRI at few days of life to evaluate the role of these exams, largely used in the *RYR1*-RCM adult and pediatric population, in an earlier age range. Indeed, only an advanced diagnostic ability could lead to adequate support and provide possible therapeutic strategies at a very young age (21–23).

## Methods

### Patients' Cohort

The cohort of *RYR1*-RCM included 17 patients who were admitted to the Neuromuscular Unit of Fondazione IRCCS Ca' Granda Ospedale Maggiore Policlinico from 2009 to 2019. Among them, four presented with congenital neonatal severe onset. Muscle biopsy was performed in the first days of life in two out to four (days 5 and 37, respectively). Muscle ultrastructural studies were carried out in two patients out of four. Two patients underwent brain MRI in the first months of life (15 days and 2 months, respectively), one of them also a fetal brain MRI. Three children performed EMG in the first week of life. Muscle MRI was performed in three out of four between 18 months and 7 years of age.

### Neurophysiologic Studies

In babies aged <2 years, nerve conduction studies were performed with specialized stimulating and recording electrodes, referring to normative age-related data for conduction velocities and parameters (24). Needle electromyography was performed with a 30-gauge needle without sedation. Deltoid, biceps brachii, tibial anterior, vastus medialis and orbicularis oris muscles were tested by spontaneous or induced activation. In three out of four neonates, EMG was performed inside a neonatal intensive care unit (NICU) setting. Exams were performed by a dedicated pediatric neurophysiologist trained to operate in the NICU setting, referring to normative data for age (24, 25).

### Muscle Histopathology and Immunofluorescence

After we had obtained written informed consent, an open muscle biopsy was performed in the quadriceps muscle in all patients. Cryosections of muscle biopsies were stained with Modified Gomori Trichrome, Hematoxylin-Eosin, ATPase, nicotinamide adenine dinucleotide (NADH), and were tested with monoclonal antibodies against candidate proteins such as α-dystroglycan, theletonin, sarcoglycan, emerin, and merosin (26). For immunofluorescence, muscle cryosections were collected on Superfrost slides, fixed with 4% paraformaldehyde for 10 min, permeabilized with 0.3% Triton X-100, incubated in blocking solution (0.5% bovine serum albumin [BSA], 10% horse serum in phosphate-buffered saline [PBS]) for 30 min, and incubated with primary antibodies at 4°C (0.5% BSA, 2% horse serum in PBS). After incubation with secondary antibodies, slides were stained with DAPI (Vector, Burlingame, CA) and examined using an optic microscope (Olympus BX60, Tokyo, Japan).

### Electron Microscopy

Specimens were fixed after surgery in 2.5% glutaraldehyde in 0.1 mol/L phosphate buffer at pH 7.2 to 7.4 and postfixed in 1% OsO_4_ in the same buffer. After dehydration, specimens were embedded in Spurr. Thin sections were then stained in uranyl acetate and lead citrate and observed in a transmission electron microscope (Philips 410T) (27).

### Brain and Muscle MRI

Muscle and fetal brain MRI were performed using a 1.5T Philips Achieva scanner. Neonatal brain MRI were performed using a 3T Philips Achieva scanner. All the exams were acquired without sedation, using feeding and wrapping strategies for the neonatal time, and with the collaboration of families for the muscle MRI, allowing either the mother or the father to go inside the scanner with their child for a short exam (maximum 10 min of acquisition) using axial Turbo Spin Echo (TSE) T1 sequences to evaluate atrophy and fatty infiltration.

### Genetic Analysis

All subjects underwent genetic analysis through Sanger sequencing or NGS. After obtaining written informed consent, patient genomic DNA was extracted from the blood sample with the FlexiGene DNA Kit (Qiagen) following the manufacture's protocol. Patient DNA analysis by NGS was performed on the Illumina MiSeq System. DNA was fragmented and the target sequences were enriched by PCR methods (HaloPlex Target Enrichment System either the SureSelect technology or Nextera). The sequence analysis was evaluated using different software, and nucleotide differences between the patient's DNA and the reference sequence were identified. Variants were sought in Ensemble and LOVD mutation database. The variant frequency was evaluated using the ExAC database and the pathogenicity was evaluated through *in silico* prediction programs (Polyphen and SIFT). Potential pathological variants were confirmed by Sanger sequencing (3130 Genetic Analyzer Applied Biosystems), according to the manufacturer's protocol. Genotype-phenotype associations were investigated through specific Databases as Pubmed, ClinVar, OMIM, and Genetable. Each new variant was analyzed for inheritance and parental segregation. When a splicing alteration was suspected, total RNA was extracted from a muscle sample (Eurogold RNA pure, Euroclone) and reverse transcribed (Transcriptor High Fidelity cDNA Synthesis kit, Roche); then cDNA was amplified and processed by Sanger sequencing.

## Results

We selected four cases of severe neonatal onset *RYR1*-RCM focusing on the early findings, the driving diagnostic elements, and the subsequent follow-up (Table 1). Most of the subjects had Italian origin, while one patient was Egyptian. In two of them, a muscle biopsy was performed in the first days of life (day 5 and 37, respectively), muscle ultrastructural studies were carried out only in two patients showing abnormalities typical of CM. Two out of four underwent brain MRI in the first months of life (15 days and 2 months, respectively), one also a fetal brain MRI. EMG was performed in three children in the first week of life, identifying myogenic potentials and excluding neurogenic conditions. Muscle MRI obtained within the first years of life shows a typical pattern of *RYR1*-CM. The diagnosis was confirmed through genetic analysis, in three out of four cases using NGS panels set up during the initial assessment. Patients I and II harbored novel variants in *RYR1*.

**Table 1:** Instrumental assessment in severe congenital neonatal *RYR1*-RCM.

**Pt**	**Genetic analysis**		**Muscle biopsy**			**EMG**		**MRI**			
								**Brain**		**Muscle**	
	**Method**	***RYR1* mutation**	**Timing**	**Method**	**Results**	**Timing**	**Results**	**Timing**	**Results**	**Timing**	**Results**
I	NGS	c.3485C>T/ c.3485C>T p.T1162I	5 days	Histology	Severe type I fibers hypotrophy	1 day	Marked reduction of recruitment; absence of spontaneous and voluntary movement	15 days	Vermis hypoplasia Optical nerve thinning Enlargement of periencephalic spaces Hemispheric subdural hematoma Slight increase of lactate peak	-	
				Oxidative enzymes	Mild cytochrome C oxidase deficiency						
				Ultrastructure	Focal areas of sarcomeric abnormalities with Z line streaming and nemaline bodies, core and core-rod elements						
II	NGS	c.14928C>G/ c.14344G>A p.F4976L/ p.G4782R	37 days	Histology	Fiber size variability	4 days	Myogenic potentials	27 GA 2 months	Enlargement of periencephalic subarachnoid spaces Dilatation of periencephalic spaces Subdural hematoma in the left hemisphere Punctate white matter confluent periventricular alterations	18 months	Fat infiltration in gluteus maximus, sartorius, adductor magnum and soleus muscle
				Oxidative enzymes	Scattered cytochrome-C oxidase activity						
III.1	NGS	c.14928C>G/ c.14928C>G p.F4976L	2 years	Histology	Type I fibers hypotrophy, fiber splittings, moderate connective tissue	5 days	Myogenic potentials, particularly in facial muscles	3 days	No abnormalities	5 years	Muscle atrophy with bilateral fat substitution of glutei and vastus, sparing of rectus femoris, adductor magnus and soleus muscles
				Ultrastructure	Z-line streaming						
III.2	Sanger	c.14928C>G/ c.14928C>G p.F4976L	3 years		Not diagnostic (connective tissue)	3 years	Myogenic potentials	-		7 years	Muscle atrophy, fat substitution of pelvic girdle muscles, thigh and lower leg muscles, sparing of rectus femoris, adductor longus, gracilis and tibialis anterioris

*Genetic analysis, muscle biopsy, electromyography (EMG), and brain and muscle Magnetic Resonance Imaging (MRI) are detailed for the four patients with severe congenital neonatal onset. Patients III.1 and III.2 are cousins*.

### Patient's Description

#### Patient I

Patient I was a female second-child, born at term (G.A. 36) from consanguineous Egyptian parents. Her sister was 2 years older and presented neither perinatal nor growing problems. From the seventh month of pregnancy the mother reported a reduction of fetal movements and polyhydramnios. At birth, the patient presented hyporeactive with severe generalized hypotonia, hypovalid crying, and peripheral acrocyanosis (APGAR 3 at 1 min). Weight was 2,150 g. Weakness distribution was axial and proximal causing the absence of spontaneous movements. Neonatal reflexes were poor. The patient presented weak tendon reflexes associated with contractures at the knees (20°). Facial muscles weakness determined open mouth and poor sucking, thus she was fed by gavage. Non-invasive ventilation (NIV) was provided during the first days of life for respiratory failure with normal diaphragmatic motility, and biphasic NIV for 15 h a day was prescribed. Serum CK levels were normal. Electroencephalogram (EEG) showed an adequate organization with minimal signs of immaturity. EMG was performed the first day of life, showing a marked reduction of muscular recruitment and absence of spontaneous or voluntary movement at deltoids, biceps brachii, quadriceps, and tibialis anterior muscles; no signs of denervation and no altered motor unit potential morphology or amplitude decrease at repetitive stimulation were detected; sensitive conduction velocities were normal.

**Figure 1:**
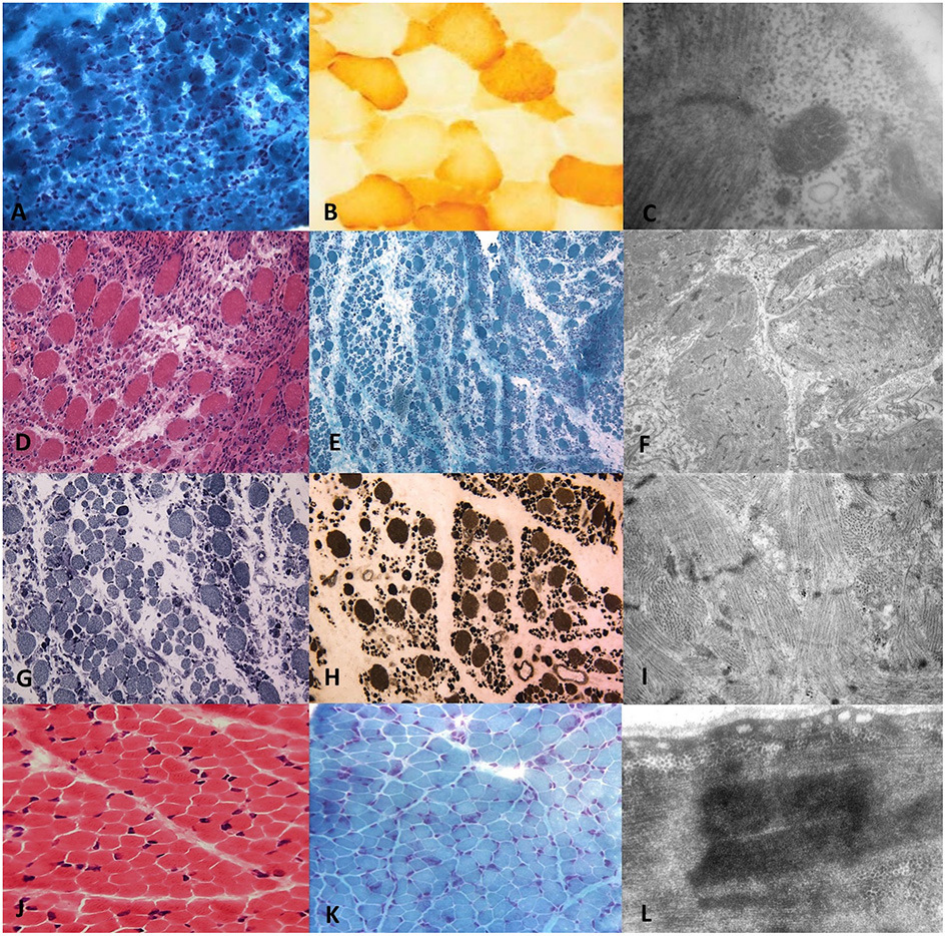
Histopathological and ultrastructural features on transverse sections of muscle biopsy of *RYR1*-RCM. Muscle biopsy from Patient I performed at 5 days of life in the first line. **(A)** Gomori Trichromic staining showed fiber size variability; **(B)** Tiny cytochrome-C oxidase deficiency at COX staining; **(C)** Central core and rod-core elements at electron microscopy. Muscle biopsy from Patient II was performed at 37 days of life in the second and third lines. **(D)** Severe type I fibers hypotrophy at Hematoxylin-Eosin, **(E)** Gomori trichromic, **(G)** NADH-TR, and **(H)** ATPase after acid preincubation showing predominance of darker staining hypotrophic type 1 fibers. **(F,I)** Electron microscopy showing focal areas of sarcomeric abnormalities with Z-line streaming and, in rare fibers, subsarcolemmal and intermyofibrillar deposits of free glycogen. Muscle biopsy from Patient III.1 was performed at 2 years of age in the fourth line. Fiber size variability with fiber I hypotrophy, fiber splittings, and moderate connective tissue increase **(J)** Hematoxylin-Eosin and **(K)** Gomori Trichromic; **(L)** Z-line streaming at electron microscopy.

At 5 days of life, the girl underwent a muscle biopsy (left quadriceps muscle) showing severe type I fibers hypotrophy and mild cytochrome C oxidase deficiency. An ultrastructural study showed focal areas of sarcomeric abnormalities with Z line streaming, central cores, and rod elements and, in rare fibers, subsarcolemmal and intermyofibrillar deposits of free glycogen (Figures 1A–C). Genetic analysis for *TK2, SURF1, SMN1, DM1*, and respiratory chain complex IV-related genes resulted negative for mutations. NGS panel (199 neuromuscular related genes) revealed the novel homozygous *RYR1* mutation c.C3485T (p.Thr1162Ile) segregating in her parents; the sister was a heterozygous carrier.

**Figure 2:**
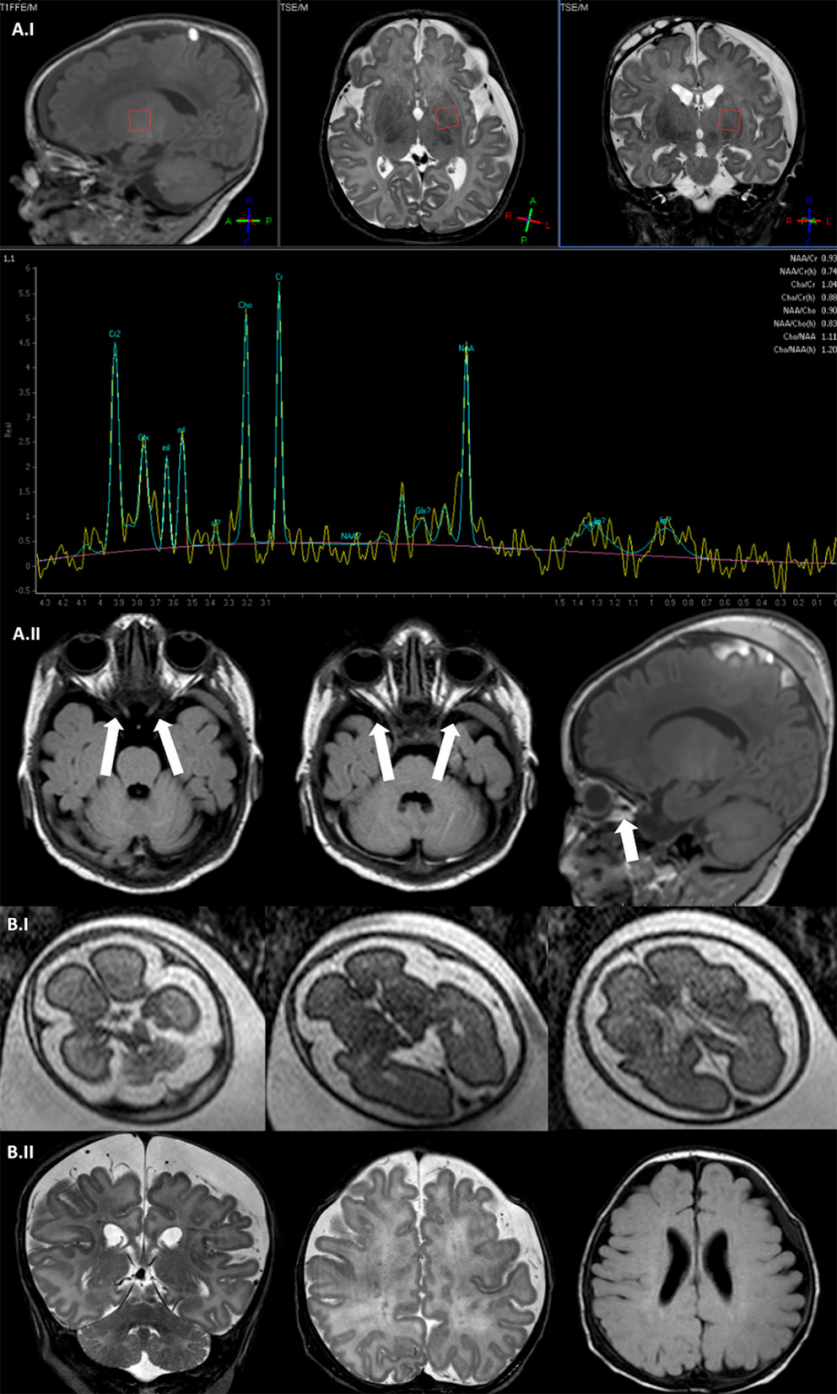
Brain MRI. **(A.I)** Fetal brain MRI (Patient II; 27 G.A.), axial SShTSE T2 images showing a bilateral enlargement of periencephalic spaces. **(A.II)** Brain MRI coronal and axial TSE T2 image (Patient II; 2 months) showing enlargement of periencephalic spaces with subdural hematoma, multiple punctate lesions correlated with prematurity in the periventricular white matter. **(B.I)** Spectral brain MRI with a single voxel = 35 ms positioned at the left basal ganglia showing a slight increase of lactate peak (Patient I, 15 days). **(B.II)** Axial and coronal TSE T2 image showing a bilateral optic nerve thinning and the subdural hematoma (Patient I, 15 days).

Brain MRI was performed at 15 days of life showing an enlargement of periencephalic spaces and a hemispheric subdural hematoma (Figure 2A.I), mild vermis hypoplasia, and optical nerve thinning (Figure 2A.II). E. Spectral brain MRI revealed a slight increase of lactate peak (Figure 2A.I). At 2 months of life, poor visual tracking was noticed and vascular papillar malformation in the left eye was identified, but visual evoked potentials were normal. Echocardiography resulted in normal cardiac function, no sign of cardiomyopathy, and Electrocardiography (ECG) showed sinus tachycardia.

At 3 months of age for inadequate feeding, a percutaneous endoscopic gastrectomy (PEG) was provided for inadequate feeding, and nocturnal non–invasive respiratory support was maintained. After the critical perinatal period, the girl progressively improved and acquired motor milestones, with some psychomotor delay. At the last follow-up visit at the age of 3 years, she acquired independent ambulation, maintained NIV during nighttime, and was fed through PEG. Visual ability recovered. She is continuing physiotherapy and logopedic program presenting improvements in language, autonomous standing, and moving steps.

#### Patient II

Patient II was a preterm female child presenting at birth (G.A. 34 weeks) presenting severe congenital floppy baby syndrome at birth. She was the first-born child of non-consanguineous parents and the family history was negative for neuromuscular diseases. Intrauterine life was remarkable for polyhydramnios (G.A. 25), suspected esophageal atresia, and micrognathia. To clarify suspected malformative features, at 27 G.A., intrauterine fetal brain MRI was performed showing an enlargement of periencephalic subarachnoid spaces, but neither malformative nor focal parenchymal brain lesions were detected (Figure 2B.I). Fetal echocardiography was normal. Comparative Genome Hybridization (CGH) array resulted negative.

At birth, the patient presented marked hypotonia with generalized weakness, hypomimia, and absence of spontaneous movement (APGAR 5 at 1 min, 6 at 5 min). Her fingers were thin. Weight at birth was 2,000 g. Invasive ventilation was required for 55 days, followed by NIV. Echocardiography showed signs of concentric hypertrophy without structural cardiomyopathy. CK serum level was in the normal range. At 4 days of life, an EMG was performed, showing myogenic polyphasic potentials (orbicularis oris, deltoids, biceps brachii, quadriceps, and tibialis anterior muscles were bilaterally tested), without amplitude decrease at repetitive stimulation. The EEG was remarkable for diffuse signs of immaturity. Genetic tests for Prader-Willy syndrome, *SMN1*, and *DM1* resulted negative.

At the age of 37 days, the patient underwent a biopsy at the right quadriceps muscle which showed fiber size variability and scattered cytochrome-C oxidase activity (Figures 1D–I).

NGS panel showed two heterozygous compound *RYR1* mutations, segregating in her parents. The maternal allele carried the novel mutation c.G14344A (p.Gly4782Arg), while the paternal allele carried the c.C14928G (p.Phe4976Leu) mutation (28).

**Figure 3:**
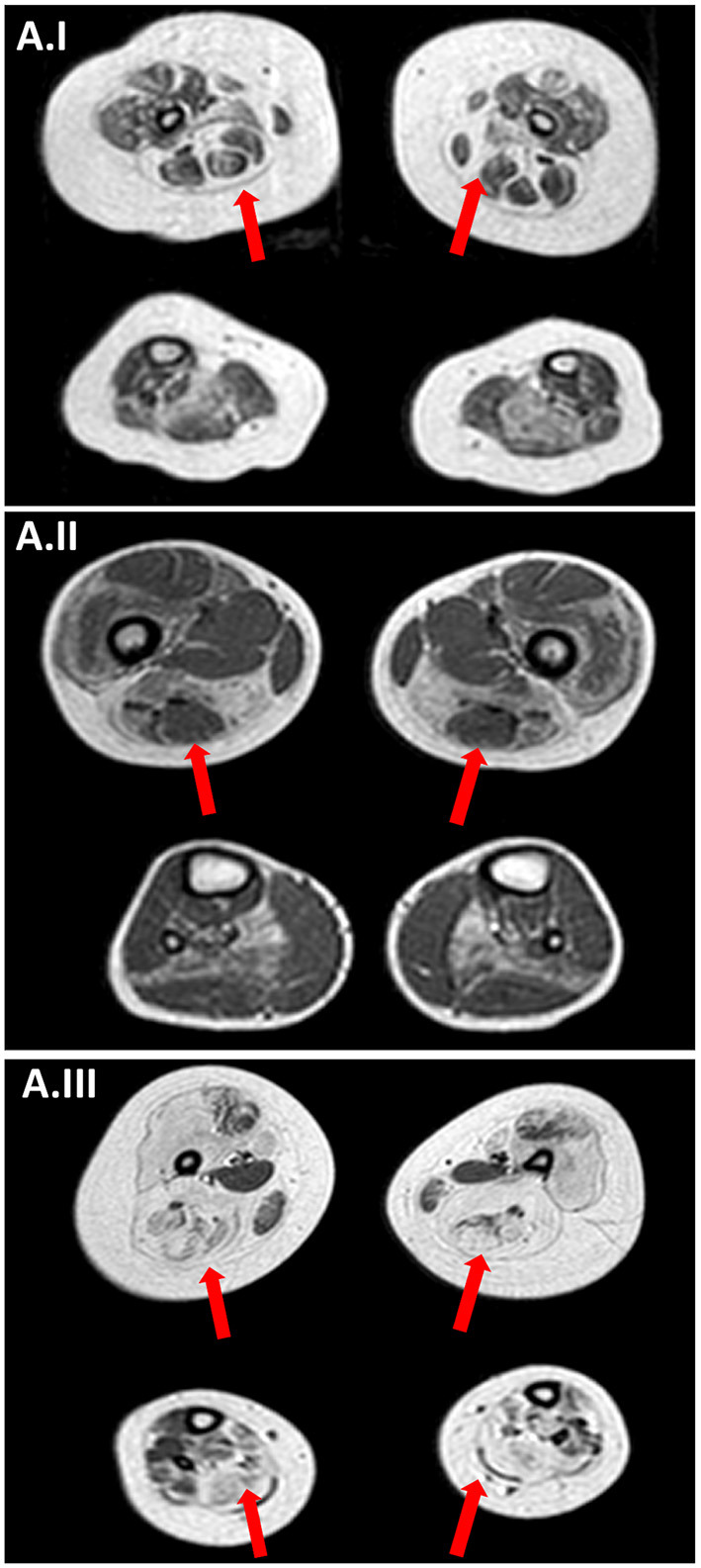
Muscle MRI. **(A.I)** Muscle MRI (Patient II; 18-months old) axial TSE T1 images at thigh and lower leg level showing a bilateral and symmetrical atrophy and fatty infiltration of gluteus maximus, sartorius, adductor magnum and, at lower leg level, in soleus muscle a pattern compatible with *RYR1*-RCM. **(A.II)** Muscle MRI (Patient III.1; 5 years old) axial TSE T1 images at thigh and lower leg level showing a bilateral and symmetrical atrophy and fatty infiltration of vastus lateralis, sartorius, adductor magnum and, at lower leg level, in soleus muscle a pattern compatible with *RYR1*-RCM. **(A.III)** Muscle MRI (Patient III.2; 7 years old) axial TSE T1 images showing bilaterally muscle atrophy with diffuse fat substitution of pelvic girdle muscles thigh and lower leg muscles, with relative sparing of rectus femoris, adductor longus, gracilis, and tibialis anterioris.

Brain MRI was repeated at 2 months of life, showing microcephaly with moderate dilatation of periencephalic spaces, subdural hematoma in the left hemisphere, in association with punctate white matter confluent periventricular alterations, expression of prematurity (Figure 2B.II). Muscle MRI (18 months of age) demonstrated atrophy and fat infiltration in gluteus maximus, sartorius, adductor magnum, and in soleus muscle, a pattern compatible with *RYR1*-RCM (Figure 3A.I). At 4 months of age, the patient required tracheostomy and PEG. In the following months, the patient showed improvements in muscle tone and strength. At 11 months of age, she could maintain the sitting position.

At the last follow-up visit, the girl was 3 years old and she could speak with moderate dysarthria, maintained the sitting and standing position with bilateral support, and upraised superior arms above shoulder level. Tendon reflexes were absent. She maintained PEG and NIV. She suffered from recurrent respiratory tract infections.

#### Patient III.1

Patient III.1 was a preterm male (37 weeks G.A.), presenting at birth with marked axial and lower limb hypotonia, absence of spontaneous movements, and respiratory failure requiring ventilation (APGAR 7 at 1 min and 8 at 5 min). Moreover, severe facial weakness with feeding difficulties, poor neonatal reflexes, and arthrogryposis with joint contractures were present. Weight was 2,790 g, height 48 cm, head circumference 37 cm. EMG performed in the first week of life showed myogenic potentials at four limbs and facial muscles and the absence of neuromuscular joint pathology signs. Brain echography the day after birth and brain MRI a few days after were negative. Serum CK levels were normal. Anti-AchR and anti-MuSK autoantibodies were absent. Karyotype was normal and genetic testing for Prader-Willi, *SMN1* and *DM1* were negative. A variant in *SCNA4* was considered not pathogenic since it was inherited from the asymptomatic mother whose EMG was negative for paramyotonia. No signs of ocular or cardiac involvement were detected. He manifested generalized weakness and hypotrophy, motor milestones were delayed and independent ambulation was acquired at 18 months of age.

A muscle biopsy was performed in the right quadriceps muscle at 2 years of age showing fiber size variability, fiber I hypotrophy, fiber splittings, and moderate connective tissue increase. Protein staining for dystrophin, emerin, merosin, and sarcoglycan was normal, while ultrastructural analysis revealed Z line streaming (Figures 1J–L). At the age of 5 years, the NGS panel (59 genes) identified a homozygous *RYR1* mutation c.C14928G (p.F4976L) (28), segregating in parents. Muscle MRI, performed at 5 years of age, showed muscle atrophy with bilateral fat substitution of glutei, vastus muscles with relative sparing of rectus femoris, adductor magnus, and soleus muscles, compatible with *RYR1*-RCM (Figure 3A.II). The patient underwent motor rehabilitation, psychomotricity, and logopedic programs. At 7 years of life, he maintained independent ambulation, presented facial weakness, but he was able to speak and did not need respiratory support.

#### Patient III.2

Patient III.2, cousin of Patient III.1, was the last of three children: one asymptomatic brother aged 7 years older and a sister deceased at 2 months of life after a pregnancy characterized by polyhydramnios and a perinatal period with hypotonia and respiratory failure.

The pregnancy of Patient III.2 was characterized by the absence of fetal movements and polyhydramnios from 35 G.A., with normal karyotype and the CGH-array from amniocentesis. At birth (preterm 35 weeks of G.A.) the patient presented bradycardia, respiratory failure requiring invasive ventilation (APGAR 1 at 1 min, 5 at 5 min, and 8 at 10 min), marked generalized hypotonia and weakness (mainly in proximal and facial muscles) associated with muscle atrophy, severe dysphagia, dysmorphic traits, and joint contractures. Weight at birth was 2,700 g, height 48 cm, head circumference 35 cm. Serum CK levels were normal. After the intensive care rescue, the patient needed NIV for 1 month and feeding through gavage, but at 3 months of life, he required PEG. He developed scoliosis and motor delay, therefore, at the age of 3-years old, the patient was evaluated by EMG which showed myogenic potentials in proximal superior and inferior limbs muscles. He underwent a muscle biopsy (quadriceps muscle) which was inconclusive because it mainly consisted of connective tissue. Echocardiography and ECG revealed no cardiomyopathy. After the genetic diagnosis of Patient III.1, *RYR1* was sequenced in Patient III.2 identifying the same homozygous mutation c.C14928G, correctly segregating. Muscle MRI performed at 7 years of age showed bilateral muscle atrophy with diffuse fat substitution of pelvic girdle muscles, thigh and lower leg muscles, with sparing of rectus femoris, adductor longus, gracilis, and tibialis anterioris (Figure 3A.III). The patient followed a rehabilitation program; from the age of 3 years old, he was free from NIV. The last clinical evaluation at 8 years of age confirmed generalized weakness, non-autonomous ambulation, scoliosis, and dysphagia requiring PEG.

## Discussion

Thanks to recent advances in genetic analysis techniques, the spectrum of *RYR1*-RCM is widening. It now comprises heterogeneous phenotypic manifestations such as perinatal life-threatening conditions with respiratory involvement and hypotonia, malignant hyperthermia susceptibility (29) and exertional heat stroke (30), rhabdomyolysis-myalgia syndrome, King-Denborough syndrome, scoliosis, club feet, arthrogryposis, hip dislocation, atypical periodic paralysis (31), and to late-onset muscular weakness. In line with the literature, cases from our cohort of *RYR1*-RCM cover this phenotype heterogeneity.

Though the severe congenital onset in the neonatal period has been remarked in various studies, few cases have been extensively characterized. Therefore, we focused on four patients and assessed onset at a very early stage of life, considering the importance of each element in the diagnostic process to define the best approach at very young ages.

Three children underwent EMG in the first week of life with identification of myogenic potentials and absence of neurogenic pathologies. Laforgia had described the early application of EMG in a severe congenital *RYR1*-RCM at 9 days of life, showing myogenic potentials (18). The role of EMG is controversial in early onset congenital myopathies. EMG is a technique that could be safely and rapidly performed bedside on the very first day of life in the setting of NICU. However, myopathies present challenges since neurophysiological experience is needed in recognizing subtle abnormal patterns in children under 2 years of age (25). In congenital myopathies, large units can present associated with short duration (due to the presence of hypertrophied muscle fibers) or long duration (neurogenic units as a consequence of muscle splitting and re-innervation). The distinction between primary neurogenic and myopathic abnormalities can be facilitated by finding short duration myogenic polyphasic units in interference patterns (25). In our experience, EMG identified early myogenic signs excluding neurogenic causes of floppy baby presentations as Spinal Muscular Atrophy or neuromuscular junction abnormalities.

Two subjects with severe congenital neonatal forms underwent muscle biopsy at 5 and 37 days of life, respectively. Muscle ultrastructural studies were carried out in two patients out of four, showing abnormalities typical of CM. In our experience and according to the literature, no CM cases are reported with muscle biopsy performed before the age of 12 days. In *RYR1*-RCM literature, muscle biopsies were performed in 20 children before 1 year of age (3, 18–20). Particularly, in the case described by Laforgia, the muscle biopsy was performed at 12 days of life (18) and 14 days of life in one patient of the Abath Neto series (20). Common histopathological features common in these cases were fiber size variability namely fiber type disproportion with type I predominance, central accumulation and core elements, mild to moderate increase in connective tissue in the absence of prominent degeneration and regeneration. Cores were common in dominant cases, while the recessive cases presented huge heterogeneity in muscle pathology (18, 19). However, the degree of histologic abnormalities did not correlate with phenotype severity (19). Correlations with muscle MRI were not provided in these studies. In our severe congenital *RYR1*-RCM cases, muscle biopsy showed heterogeneity of findings which varied from fiber size variability, rounded fibers, and type I fiber atrophy. Interestingly, the two patients who underwent muscular biopsy very precociously showed misleading aspects, leading to the hypothesis of mitochondrial disease in Patient I, and of SMA in Patient II. However, even in these cases, addressing features were detected at electron microscopy, suggesting the utility of its implementation. On the other side, considering the high variability of muscle biopsy features in the first years of life, the NGS study should be proposed as the first diagnostic tool in cases with a high suspicion of CM.

In literature on this subject, the earliest muscle biopsy-MRI comparison regarded three female patients in which muscle biopsy was performed, respectively, at 6-months, 2, and 2.5-years of age, respectively (20). Muscle biopsies of these patients showed marked fiber size variability, fiber type disproportion, and intermyofibrillar abnormalities (this feature reported only in the last one), while muscle MRI showed severe generalized muscle substitution in the first patient, very mild muscular involvement in the second, and marked impairment of vastus lateralis, sartorius and adductor magnus muscles in the oldest patient (20). Despite some degree of variability in severity, the vastus lateralis was the most involved muscle while the rectus femoris resulted generally spared (20). Muscle MRI in pediatric *RYR1*-RCM was described also by Klein in five autosomal recessive cases and one autosomal dominant, all presenting the same pattern of distribution despite heterogeneity in the severity of the muscular involvement (6).

Muscle MRI obtained within the first years of life shows a typical pattern of *RYR1*-CM. Muscle MRI confirmed the *RYR1*-RCM typical pattern of distribution even in the very early stage of the disease (16, 17), which is characterized by predominant involvement of gluteus maximus, adductor magnus, vastus, and soleus muscles with relative sparing of rectus femoris and adductor longus. Moreover, muscle MRI is a relatively fast exam, because axial T1 images are enough to evaluate atrophy and fatty infiltration. In our opinion, with this technique, such muscular pathological condition is identifiable since the onset. Furthermore, the concern of sedation required for performing muscle MRI in infants is less relevant in these patients since they are often ventilated due to relation to respiratory failure. The head of the patient also remains outside the scanner gantry, so it is easier to obtain the child's compliance, particularly because the parents could get into the scanner room as well.

Our collection presented the unique images of intrauterine brain MRI in *RYR1*-RCM. Two out of four neonatal severe cases underwent brain MRI in the first months of life (15 days and 2 months, respectively), one also a fetal brain MRI. Bilateral optic nerve thinning and the enlargement of periencephalic subarachnoid spaces are not specific malformative features of the condition but we reported them in both patients who underwent brain MRI. The white matter confluent periventricular alterations described in one case were an expression of prematurity. Even if CNS involvement is not a prominent element in *RYR1*-RCM, data regarding this information related to the phenotype are lacking.

The diagnosis was confirmed through genetic analysis starting from NGS panels set up during the initial assessment in three out of four neonatal cases. Since the final diagnosis is genetically defined and due to the high diagnostic rate of extensive sequencing techniques (32), we suggest including the diagnostic approach at an early stage for similar cases. In an NGS study with a wide panel of genes related to neuromuscular congenital conditions or exome sequencing, a huge number of variants could emerge and the instrumental studies here discussed are important in the interpretation of the genetic results.

Regarding the neurological follow-up of these children, after the intensive care rescue, they survived and demonstrated a stable or even ameliorative course of the disease since no patients required further invasive ventilation and two of them acquired independent ambulation. Enteral nutrition is a major topic since facial weakness is marked in all severe congenital patients.

In conclusion, deep phenotyping and genotyping are fundamental for reaching a correct diagnosis and deepening knowledge on these rare disorders. This should become even more relevant since it may lead to immediate medical management and it helps to optimize inclusion in future clinical trials.

## Data Availability Statement

Publicly available datasets were analyzed in this study. This data can be found here: SCV001653512.1 RCV001449965.1 NM_000540.2: c.3485C>T SCV001653513.1 RCV001449966.1 NM_000540.2:c.14344G>A;NM_000540.2:c.14928C>G SCV001653514.1 RCV001449967.1 NM_000540.2:c.14928C>G.

## Ethics Statement

The studies involving human participants were reviewed and approved by Comitato Etico Milano Area 2. Written informed consent to participate in this study was provided by the participants' legal guardian/next of kin. Written informed consent was obtained from the minor(s)' legal guardian/next of kin for the publication of any potentially identifiable images or data included in this article.

## Author Contributions

EM and FM conceived the idea, revised the literature, and wrote the manuscript. DP, SP, DC, and VN performed the genetic analysis and interpreted the results. MR and MS analyzed and interpreted muscle histopathologic and electron biopsy imaging. RD performed neurophysiologic studies and interpreted the results. CC performed and interpreted muscle and brain MRI data. AG, RB, SC, GC, and NB performed a critical revision of the manuscript for important intellectual content. All authors contributed to manuscript revision and read, approved the submitted version, and took care of patients' management and decisions.

## Conflict of Interest

The authors declare that the research was conducted in the absence of any commercial or financial relationships that could be construed as a potential conflict of interest. The Handling Editor declared a past co-authorship with several of the authors VN, DC, GC, and FM.
